# Profiling Docetaxel in Plasma and Urine Samples from a Pediatric Cancer Patient Using Ultrasound-Assisted Dispersive Liquid–Liquid Microextraction Combined with LC–MS/MS

**DOI:** 10.3390/pharmaceutics15041255

**Published:** 2023-04-17

**Authors:** Olga Maliszewska, Anna Roszkowska, Marcin Lipiński, Natalia Treder, Ilona Olędzka, Piotr Kowalski, Tomasz Bączek, Ewa Bień, Małgorzata Anna Krawczyk, Alina Plenis

**Affiliations:** 1 Department of Analytical Chemistry, Medical University of Gdansk, 80-416 Gdańsk, Poland; 2 Department of Pharmaceutical Chemistry, Medical University of Gdansk, 80-416 Gdańsk, Poland; 3 Department of Pharmaceutical Biochemistry, Medical University of Gdansk, 80-211 Gdańsk, Poland; 4 Department of Pediatrics, Hematology and Oncology, Medical University of Gdansk, 80-211 Gdańsk, Poland

**Keywords:** docetaxel, plasma sample, urine sample, ultrasound-assisted dispersive liquid–liquid microextraction (UA-DLLME), liquid chromatography with tandem mass spectrometry (LC–MS/MS)

## Abstract

In recent years, therapeutic drug monitoring (TDM) has been applied in docetaxel (DOC)-based anticancer therapy to precisely control various pharmacokinetic parameters, including the concentration of DOC in biofluids (e.g., plasma or urine), its clearance, and its area under the curve (AUC). The ability to determine these values and to monitor DOC levels in biological samples depends on the availability of precise and accurate analytical methods that both enable fast and sensitive analysis and can be implemented in routine clinical practice. This paper presents a new method for isolating DOC from plasma and urine samples based on the coupling of microextraction and advanced liquid chromatography with tandem mass spectrometry (LC-MS/MS). In the proposed method, biological samples are prepared via ultrasound-assisted dispersive liquid–liquid microextraction (UA-DLLME) using ethanol (EtOH) and chloroform (Chl) as the desorption and extraction solvents, respectively. The proposed protocol was fully validated according to the Food and Drug Administration (FDA) and the International Council for Harmonization of Technical Requirements for Pharmaceuticals for Human Use (ICH) requirements. The developed method was then applied to monitor the DOC profile in plasma and urine samples collected from a pediatric patient suffering from cardiac angiosarcoma (AS) with metastasis to lungs and mediastinal lymph nodes, who was receiving treatment with DOC at a dose of 30 mg/m^2^ body surface area. Due to the rarity of this disease, TDM was carried out to determine the exact levels of DOC at particular time points to ascertain which levels were conducive to maximizing the treatment’s effectiveness while minimizing the drug’s toxicity. To this end, the concentration-time profiles of DOC in the plasma and urine samples were determined, and the levels of DOC at specific time intervals up to 3 days after administration were measured. The results showed that DOC was present at higher concentrations in the plasma than in the urine samples, which is due to the fact that this drug is primarily metabolized in the liver and then eliminated with the bile. The obtained data provided information about the pharmacokinetic profile of DOC in pediatric patients with cardiac AS, which enabled the dose to be adjusted to achieve the optimal therapeutic regimen. The findings of this work demonstrate that the optimized method can be applied for the routine monitoring of DOC levels in plasma and urine samples as a part of pharmacotherapy in oncological patients.

## 1. Introduction

Docetaxel (DOC) is a semisynthetic antineoplastic agent belonging to the taxane family that is obtained via the chemical modification of natural substances from the Yew tree (*Taxus bcc.*) [1]. DOC’s main mechanism of action is to disrupt the function of microtubules. Specifically, DOC causes the hyper-stabilization of the microtubules’ structure which, in turn, inhibits the function of cancer cells and ultimately results in their death due to blockage of the cell cycle [2]. DOC is widely used as a single agent or as part of polytherapy in the treatment of various cancers, such as locally advanced or metastatic breast cancer, metastatic prostate cancer, gastric adenocarcinoma, and head and neck cancer [3]. DOC is also used in the treatment of numerous pediatric cancers, including osteosarcoma [4], the Ewing sarcoma family of tumors (ESFT) [5], and cardiac angiosarcoma (AS), which is one of the rarest childhood cancers [6]. During chemotherapy, DOC is administered intravenously (IV) at doses ranging between 60 and 100 mg/m^2^ for 1 h every 3 weeks [7].

Studies have found that the pharmacokinetics of DOC are best described using a three-compartment model, with half times of 4–5 min, 38.3 min, and 12.2 h [8]. Moreover, DOC strongly binds to plasma proteins (~95%), with 75% and 5% of administered doses being eliminated from the body via feces and urine, respectively [9]. The main enzyme involved in DOC metabolism, P450 (CYP3A4), often interacts with other drugs, which can intensify the adverse effects of DOC treatment, including neutropenia, diarrhoea, alopecia, and inflammation of the mucous membranes [8]. Furthermore, previous research has shown that pharmacokinetics data, such as plasma concentrations or area under the curve (AUC), correlate with adverse effects of DOC treatment. For example, the optimal AUC values for Asian patients was determined to be 2.5–3.7 μg·h/mL (for a dose of 75 mg/m^2^ every 3 weeks) [10]. On the other hand, according to previous research, selected AUC values did not exceed 4.9 mg·h/L in the case of a 75 mg/m^2^ dose every 3 weeks [11]. In addition, DOC has a narrow therapeutic window and high inter-individual variability, which necessitates constant monitoring of its concentration in biological samples, such as plasma and urine, during treatment. This is especially important in the treatment of pediatric patients, as they possess different pharmacokinetics of DOC than adults have. However, the precise analysis of DOC levels during the pharmacotherapy depends on the availability of methods capable of accurately quantifying its concentration in complex matrices. 

Since being approved for use in chemotherapy by the Food and Drug Administration (FDA) in 1996, researchers have developed numerous analytical methods to monitor DOC in various animal and human matrices, including blood and its fractions, saliva, urine, and feces [1]. While the majority of procedures for the analysis of DOC developed since 2010 have been based on liquid chromatography (LC) [12,13,14,15,16,17,18,19] and ultra-performance liquid chromatography (UPLC) [20], methods based on capillary electrophoresis (CE) [21] and immunoassays have also been reported [22]. In these works, the complex biological matrices containing DOC are subjected to sample preparation prior to instrumental analysis, mainly via classical extraction techniques such as liquid–liquid extraction (LLE) [12,13,19], protein precipitation (PPt) [14,15,23] and solid-phase extraction (SPE) [16,17,18]; however, to the best of our knowledge, microextraction techniques have yet to be applied to isolate DOC from biological matrices. This gap in the literature is notable, as microextraction techniques use significantly less hazardous chemicals, thus aligning them with the principles of green chemistry. One example of a novel and environmentally friendly microextraction technique is dispersive liquid–liquid microextraction (DLLME). In DLLME, an appropriate mixture of extraction and disperser solvents is rapidly injected into an aqueous sample solution which, in turn, forms a cloudy solution. Equilibrium between the extraction solvent and aqueous sample is reached very quickly, and fine droplets of extraction solvent enriched with the targeted analytes are collected for further analysis. Initially, DLLME was mainly applied for the analysis of water and environmental samples [24] and rarely in the analysis of complex biological matrices, such as plasma and urine [25]. The reason for this is that complex biological samples are more demanding as precipitate frequently occurs during standard sample handling, which can impede or even preclude further analysis. However, subsequent advances in DLLME have enabled its application in a broad range of areas, including the analysis of compounds in biological matrices. In addition, researchers have developed new forms of DLLME, including ultrasound-assisted DLLME (UA-DLLME), which has further expanded its range of possible uses in the bioanalysis of drugs [26].

This work presents the development of UA-DLLME coupled to liquid chromatography with the tandem mass spectrometry (LC–MS/MS) method capable of precisely isolating and quantifying DOC in human plasma and urine. To this end, various chromatographic conditions for MS/MS detection and extraction procedures based on DLLME were first tested. Next, the optimized UA-DLLME-LC–MS/MS method was validated in accordance with the FDA [27] and International Council for Harmonisation of Technical Requirements for Pharmaceuticals for Human Use (ICH) [28] guidelines. Finally, the developed method was applied to quantify DOC in plasma and urine samples obtained from a 12-year-old male cancer patient undergoing DOC-based chemotherapy via IV administration.

## 2. Materials and Methods

### 2.1. Chemicals

Docetaxel (DOC), paclitaxel (PAC) (internal standard (IS)), and formic acid (FA) were obtained from Sigma-Aldrich Co. (St. Louis, MO, USA), while LC-MS-grade acetonitrile (ACN) and methanol (MeOH) were purchased from Supelco (Darmstad, Germany). Ethanol (EtOH), analytical-grade chloroform (Chl), hydrochloric acid (36%), and sodium hydroxide were purchased from POCH (Gliwice, Poland), and dichloromethane (DCHM) was provided by Merck (Darmstadt, Germany). The water used in this work was purified using a Mili-Q system (Molshem, France), and the phosphate-buffered saline (PBS) (PBS Stock Solution 10X) was obtained from the Cayman Chemical Company (Washtenaw County, MI, USA); 0.5- and 1-mL Hamilton syringes were purchased from Sigma-Aldrich Co. (St. Louis, MO, USA). All samples were sonicated in the ultrasonic bath Ultron U-504, which was purchased from Ultron (Dywity, Poland). Stock standard solutions of DOC and PAC (IS) were prepared independently by dissolving 1 mg of each compound in 1 mL of MeOH. Next, the stock solutions were dissolved in MeOH to obtain working solutions with a concentration of 100, 10, and 1 µg/mL, as well as 100 ng/mL. All standard solutions of DOC and PAC were stored in the dark at −20 °C. 

### 2.2. Sample Collection

Plasma and urine samples were collected from healthy volunteers and used during the optimization of sample preparation procedure and validation study. Moreover, plasma samples obtained from a 12-year-old male patient were collected at the following time intervals: prior to the DOC infusion (0 h); halfway through the DOC infusion (0.5 h); at the end of the DOC infusion (1 h); and 1 h, 2 h, 3 h, 4 h, 6 h, 8 h, 12 h, 24 h, 48 h, and 72 h after the DOC infusion. For the infusion, DOC was administered intravenously (IV) at a dose of 45 mg for 1 h (30 mg/m^2^).

In contrast, the urine samples were collected prior to the infusion (0 h); at the end of the infusion (1 h); and at 4.5 h, 6.5 h, 8.5 h, 9.5 h, 15.5 h, 21 h, and 72 h after the infusion. All samples were centrifuged for 10 min at 9000 rpm and stored at −80 °C in the dark until LC–MS/MS analysis.

### 2.3. LC–MS/MS Analysis

The LC–MS/MS analysis was performed using a Thermo Finnigan Surveyor high-performance liquid chromatography system that was linearly connected with a Thermo Finnigan LCQ Vantage triple quadrupole mass spectrometer. An autosampler was used to inject 2 µL of sample into a Phenomenex C-18 Kinetex 1.7 µM 50 × 2.1 mm column prefaced by a C-18 4 × 2 mm Phenomenex guard column. In this work, a mobile phase gradient consisting of two buffers was used. Buffer A consisted of 0.1% FA in distilled water, while buffer B was an organic phase comprised of 0.1% FA in ACN. The mobile phase gradient utilized a flow rate of 200 µL/min and the following schedule: 50% B at 0 min; increase from 50% to 95% B from 0 min to 2.2 min; and equalization at 50% B from 2.2 min to 3.5 min. Data were collected in positive ionization in MS/MS mode. DOC detection was conducted based on the product ion produced by a parent ion (808.2 *m*/*z*) for frequencies of 181.9 *m*/*z* and 308.80 *m*/*z*, respectively, with the collision energy being optimized at 15% for the product ion and 16% for the parent ion. Detection of PAC (854.2 *m*/*z*), which was used as the IS, was provided for 285.7 *m*/*z* and 509.2 *m*/*z* ions at 13% and 7% collision energy, respectively. Finally, a spray voltage of 4 kV, sheath gas flow at 15 arb., auxiliary gas flow at 5 arb., and a capillary temperature of 250 °C were used for the analysis.

### 2.4. Preparation of Plasma and Urine Standards

Calibration samples (CSs) were prepared by adding the working solutions of DOC and IS to blank plasma (0.5 mL) and urine (1 mL) samples. The calibration curves for the plasma samples were constructed using CSs with DOC concentrations of 2.5–2000 ng/mL and IS at 100 ng/mL, while the calibration curves for urine were constructed by spiking urine with DOC at 5–2000 ng/mL and IS at 50 ng/mL. Quality control samples (QCs) for plasma and urine were prepared at low (LQC; plasma: 50 ng/mL; urine: 250 ng/mL), medium (MQC; both matrices: 750 ng/mL), and high (HQC; both matrices: 1500 ng/mL) levels, while the IS was added to the plasma and urine samples at a concentration of 100 and 50 ng/mL, respectively. 

### 2.5. DLLME Procedure

Next, 0.5 mL of plasma or 1 mL of urine was spiked with IS at 100 and 50 ng/mL, respectively, and placed in a 5 mL Eppendorf tube and mixed gently for 5 min to evenly distribute the analytes throughout the sample. For the plasma sample, 0.5 mL of PBS was also added. Next, 1 mL of a mixture comprised of extraction solvent (400 µL of Chl) and disperser solvent (600 µL of EtOH) was quickly injected into the sample solution using a 1 mL Hamilton syringe, which facilitated the rapid formation of a cloudy solution containing many dispersed fine droplets of Chl. The resultant emulsion was sonicated for 30 s, chilled at −80 °C for 3 min, and then centrifuged at 4300 rpm for 7 min to separate the phases. The chloroform phase was collected using a 500 µL Hamilton syringe and evaporated to dryness for 30 min at 45 °C under vacuum conditions using a CentriVap (Labconco, Kansas City, MI, USA) vacuum concentrator. The resultant residue was then reconstituted with 50 µL of an ACN:water:FA (80:20:0.1, *v*/*v*/*v*) mixture and subjected to LC–MS/MS analysis.

### 2.6. Validation of Analytical Methods

The developed UA-DLLME-LC–MS/MS protocol was validated in accordance with FDA [27] and International Conference of Harmonisation (ICH) guidelines [28] with respect to selectivity, linearity, accuracy, precision, limits of detection (LOD), limits of quantification (LOQ), stability, and carry over effects. The optimization experiments for the DLLME sample-preparation procedure were performed using phosphate-buffered saline (PBS) spiked with DOC and IS at a concentration of 1 µg/mL, while the validation experiments for the optimized method were conducted using plasma and urine samples obtained from healthy volunteers.

### 2.7. Application for DOC Profiling in Real Plasma and Urine Samples

The developed DLLME-LC MS/MS method was applied for the determination of DOC concentrations in real human plasma and urine samples. This study was approved by the Bioethics Committee of the Medical University of Gdansk (Gdansk, Poland) (Nos. NKBBN/232/2015 and NKBBN/232-219/2021), and written consent was obtained from the patient and his parents prior to the collection of blood and urine samples. The patient was a 12-year-old boy in the Department of Pediatrics, Hematology and Oncology at the University Clinical Center in Gdansk, who had been diagnosed with AS of the heart with metastasis to the lungs and mediastinal lymph nodes. The first stage of the patient’s treatment regimen consisted of three cycles of CWS—chemotherapy CEVAIE (Carboplatin, Etopozid, Vinkristin, Actinomycin-D, Ifosfamid). Following new recommendations, this was supplemented by additional CWS—chemotherapy wherein VAC (Vinkristin, Adriamycin, Cyklofosfamid) is alternated with PAC. However, the patient experienced an anaphylactic reaction after the first round of PAC, so DOC was introduced as a substitute and the oncological therapy was continued. During the treatment, DOC was administered intravenously at a dose of 45 mg for 1 h (30 mg/m^2^). Additionally, the patient underwent a premedication regimen using dexamethasone and clemastine, and omeprazolum (2 × 20 mg) and Sertraline (1 × 25 mg) were also administered during the DOC treatment.

## 3. Results and Discussion

The monitoring of DOC during anticancer treatment is highly recommended, as its use can result in numerous side effects, especially neutropenic fever, and individual responses to its use in treatment are characterized by high variability, which can lead to problems with respect to toxicity [11]. Hence, TDM is recommended during DOC-based therapy, as it can maximize the effectiveness of the pharmacotherapy while minimizing the drug’s toxicity. The TDM of DOC is particularly important in the case of rare childhood cancers, as pharmacokinetic data relating to the use of this drug in pediatric cancer treatment are limited, as is information relating to effective treatment schedules [29]. According to the literature and clinical data, cardiac AS is often characterized by bad prognoses and frequent relapses using the current strategies, which involves the application of polytherapy which is challenging and problematic [30,31]. Some reports have documented issues related to treatments based on systematic chemotherapy schedules coupled with radiotherapy and surgery resection [32]. In recent years, findings have shown that the application of DOC-based therapy alongside radiotherapy can yield successful results in the treatment of cardiac AS in adults [31]. However, it should be emphasized that there are no data relating to the use of this approach in the treatment of oncologic pediatric patients; nor are there data relating to the monitoring of DOC levels in such treatments.

Despite the widespread use of DOC in anticancer therapy, the most frequently reported analytical methods for quantifying DOC in plasma and urine samples have been based on traditional approaches. Over the last 13 years, only four publications have described the determination of DOC in human plasma samples [15,17,19,23], with only one detailing the optimization of a method for extracting DOC from urine [19]. However, data relating to DOC profiles in real human urine samples remain absent from the literature. Despite this gap, the literature does contain multiple studies documenting analytical procedures for the quantification of DOC in biofluids from animals [12,13,14,16].

To the best of our knowledge, the present study is the first to propose a microextraction method consisting of UA-DLLME coupled with LC–MS/MS for the fast and precise monitoring of DOC in real plasma and urine samples.

### 3.1. Optimization of UA-DLLME-LC–MS/MS Conditions

To date, most LC methods developed for the analysis of DOC have been based on ultraviolet (UV) detection at wavelengths ranging between 227 and 230 nm [12,13,14,15,16], although MS/MS methods have also been used for this purpose [17,18,19,23]. In this study, several parameters related to the LC separation and MS/MS detection of DOC and PAC (IS) were optimized. The first step in optimizing the LC conditions was to select a suitable analytical column. According to the literature, C18 is the most popular stationary phase used for the separation of DOC [12,13,16,17,19,23]; however, C8 [14,15,18] has also been used in some studies. We tested two chromatographic columns—the Phenomenex C-18 Kinetex (1.7 µM 50 × 2.1 mm) and the Phenomenex Hydro RP 100A Synergi (2.5 µM 50 × 2 mm). Ultimately, the Phenomenex C-18 Kinetex column was selected as it provided superior retention and resolution, as well as signals that were sharper and more symmetrical. Next, tests were conducted to identify the most suitable IS. According to the data in the literature, PAC is most commonly used as an IS in the analysis of DOC [13,14,17,23], although some studies have used celecoxib instead [12]. PAC was selected as the IS for use in this study, as its physicochemical properties and behavior in extraction and chromatographic conditions are comparable to those of DOC (both substances have similar chemical structure, Figure S1). Moreover, oncological therapy protocols never use both DOC and PAC, as the combined use of these drugs can result in serious side effects due to their very similar anticancer activity. Consequently, PAC is often used as an IS during the determination of DOC. Having selected the optimal IS, we turned our attention to ascertaining the most appropriate mobile phase composition. To this end, ACN with 0.1% FA and MeOH with 0.1% FA were tested at different gradient settings, with the results indicating that ACN provided better results. Furthermore, the method’s run time (3.5 min) was shortened compared to previous LC–MS/MS methods, which used run times exceeding 5 min [18,19,23]. 

In the next stage of method optimization, several factors affecting the method’s extraction efficiency were examined. As noted above, since 2010, methods designed for the isolation of DOC from human bodily fluids have strictly been based on conventional extraction procedures. The LLE-based methods used in the past typically used solvents such as diethyl ether [12] or a mixture consisting of n-hexane:isoamyl alcohol (97:3, *v*/*v)* [13], while the PPt-based methods all used ACN for the extraction of DOC [14,15]. Conversely, the SPE-based methods typically used extraction phases consisting of nanofibers [16], hybrid phases [17], or C8 cartridges [18]. Notably, the literature contains no reports of the use of microextraction techniques for the determination of DOC in human bodily fluids. In this study, we fill this gap in the literature by developing a UA-DLLME-based microextraction technique to enable the efficient isolation of DOC and PAC from complex biological matrices. The proposed UA-DLLME protocol is distinguished from more conventional techniques, such as LLE, by a number of important features, including easy and fast sample preparation, shorter assay times, and reduced use of organic solvents. To obtain optimal extraction conditions, and to reduce precipitate formation, we tested various parameters affecting the method’s extraction efficiency, including the type and volume of extraction and disperser solvents, as well as the sonication time. 

Selecting the optimal extraction and disperser solvents is the most important step in developing the DLLME procedure. The optimal extraction solvent for DLLME should possess several features, including low solubility in water, high affinity to analytes, and higher density and lower miscibility than the aqueous phase. Conversely, the disperser solvent should be miscible with both the extraction solvent and aqueous solution to enable the dispersion of the extraction solvent into small droplets in the aqueous phase. In this study, two extraction solvents—namely, Chl (density, 1.48 g/mL) and DCHM (density, 1.32 g/mL)—were tested. In addition, three disperser solvents, MeOH, EtOH, and ACN, were tested. The volume of each disperser solvent varied from 600 to 800 µL, while the volume of each extraction solvent varied from 200 to 400 µL. These volume ranges were selected based on the results of our previous work, wherein it was confirmed that lower and/or higher volumes of disperser and extraction solvents result in scant droplet formation or insufficient separation of the phases, respectively [33]. The results showed that no droplets formed when using solutions consisting of DChM/ACN and Chl/ACN. Thus, these variants were excluded from further analysis. The extraction recoveries for DOC and PAC enabled by the remaining mixtures are shown in Table 1. The results indicated that the lowest recoveries were observed for the DChM/MeOH mixture at a ratio of 2:8 (*v*/*v*). For the DChM/EtOH mixture, better recovery was observed at a ratio of 2:8 (*v*/*v*) compared to ratios of 3:7 (*v*/*v*) and 4:6 (*v*/*v*). In the case of the Chl/MeOH mixture, the 2:8 (*v*/*v*) ratio only provided higher recoveries than the 4:6 (*v*/*v*), while similar recoveries were observed for the 2:8 (*v*/*v*) and 3:7 (*v*/*v*) for the Chl/EtOH mixture. Ultimately, the best extraction recoveries were obtained using a mixture consisting of 400 µL of Chl and 600 µL of EtOH, as lower volumes of Chl and higher volumes of EtOH resulted in insufficient extraction recovery for DOC.

**Figure 1:**
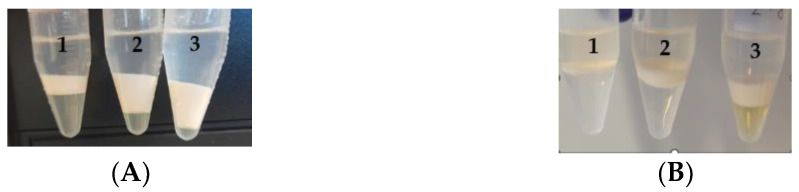
(**A**) Plasma samples after extraction via UV-DLLME with Chl:MeOH at ratios of (1) 4:6 (*v*/*v*), (2) 3:7 (*v*/*v*), and (3) 2:8 (*v*/*v*). (**B**) Plasma samples after extraction via DLLME with Chl:EtOH at ratios of (1) 4:6 (*v*/*v*), (2) 3:7 (*v*/*v*), and (3) 2:8 (*v*/*v*).

To verify the UA-DLLME method’s performance in biological samples, the two best combinations of organic solvents (Chl:MeOH and Chl:EtOH) were applied at different ratios (4:6, 3:7, 2:8, *v*/*v*) for the isolation of DOC and PAC from plasma samples. In these experiments, 0.5 mL of plasma was diluted with 0.5 mL of PBS and subjected to UA-DLLME according to the protocol described in Section 2.5. As shown in Figure 1A,B, when applied at a ratio of 2:8 (*v*/*v*), both variants of the tested solutions (Chl/MeOH and Chl/EtOH) formed droplets that were far too small to collect; as such, this ratio was excluded from use in subsequent studies. The results also showed that the Chl:EtOH mixture at a ratio of 4:6 (*v*/*v*) produced droplets with proper volume and a smaller layer of proteins at the phase boundary (Figure 1B) compared to the Chl:MeOH mixture at the same ratio (4:6, *v*/*v*) (Figure 1A). Overall, the results indicated that the mixture of Chl:EtOH at a ratio of 4:6 (*v*/*v*) provided the best extraction efficiency of both analytes in the plasma samples.

Another important parameter that may affect the extraction efficiency of analytes in UA-DLLME is the time of sonication. To test this parameter, plasma samples were enriched with DOC at the concentration of 1 µg/mL and PAC at the concentration of 100 ng/mL and were prepared according to the description of the DLLME procedure in Section 2.5, but using different times of sonification (without sonification, 0.5 min, 1 min, 2 min, 5 min of sonification). The obtained results indicated that the highest extraction efficiency was obtained for 0.5 min of sonication (Figure S2). Slightly lower extraction efficiency was found for 1 min of sonication. It should also be highlighted that a significant decrease in the extraction efficiency was observed for 2 min or longer sonication time, which may indicate progressive degradation of both DOC and PA in the samples. In addition, RSD values for 2 and 5 min sonication were above 15% for each analyte. Based on the obtained results, 0.5 min of sonication was finally selected for further analysis with the use of the proposed UA-DLLME protocol. Additionally, extraction time was optimized during the experiments on plasma samples containing DOC at the concentration of 1 µg/mL and PAC at the concentration of 100 ng/mL—which were prepared according to the description in Section 2.5 DLLME procedure—and the samples were placed at −80 °C for 1, 2, 3, 4, and 5 min. The obtained results indicated that the extraction efficiency for both DOC and PAC was increased from 1 to 3 min, and it was at the comparable level for the samples kept for 4 and 5 min in those conditions. Based on the obtained data, the extraction time of 3 min was selected as the most optimal for the analyzed compounds.

The extraction efficiency of the optimized UA-DLLME method was also tested on urine samples (1 mL of urine without dilution with PBS containing DOC and PAC at 1 µg/mL) at three different pH values (3.0, 7.0, and 10.0). For these experiments, the sample pH was adjusted by adding 0.1 M hydrochloric acid or 0.1 M sodium hydroxide (n = 3 for each tested sample pH), and the samples were subjected to UA-DLLME-LC–MS/MS analysis using the procedure described in Section 2.5, followed by analysis using the method described in Section 2.3. The results indicated that changing the pH did not affect the extraction efficiency (data not shown), so subsequent experiments on urine samples were performed at a neutral pH without the addition of an acid or base. 

In summary, the optimization experiments revealed that adding 1 mL of a Chl:EtOH (4:6, *v*/*v*) mixture to 1 mL of sample solution (0.5 mL of plasma + 0.5 mL of PBS or 1 mL of urine) provided minimal ballast precipitation and good extraction recovery for DOC and the IS in urine and plasma samples. Thus, this combination was selected for use in the UA-DLLLME sample-preparation protocol prior to LC–MS/MS analysis. Next, the optimized UA-DLLME-LC–MS/MS method was validated and applied to monitor DOC levels in a pediatric patient undergoing DOC-based chemotherapy.

### 3.2. Method Validation

Validation studies were based on the analysis of plasma and urine samples collected from healthy volunteers.

#### 3.2.1. Selectivity

According to the FDA and ICH requirements, the developed method’s selectivity was controlled by comparing blank plasma and urine samples with samples enriched with DOC at 250 ng/mL, IS at 100 (plasma), and 50 ng/mL (urine). The obtained chromatograms (Figure S3) indicate a lack of interference in the blank plasma and urine samples at retention times for DOC and IS. This confirms the selectivity of the developed UA-DLLME-LC–MS/MS protocol.

#### 3.2.2. Linearity

The developed method’s linearity for the determination of DOC in biological samples was tested by analyzing six series of calibration samples enriched with working standard solutions of DOC with analyte concentrations of 2.5, 10, 50, 100, 250, 500, 750, 1000, 1500, and 2000 ng/mL for plasma and 5, 10, 50, 100, 250, 500, 750, 1000, 1500, and 2000 ng/mL for urine, with IS concentrations of 100 ng/mL and 50 ng/mL for plasma and urine, respectively. The sample preparation and chromatographic separation of the plasma and urine CSs were conducted according to the protocols described in Section 2.5 and Section 2.3, respectively, with the DOC concentrations being calculated based on the corresponding calibration curve. These calibration curves were established on the basis of the analysis of six series of the calibration samples prepared in the range of 2.5–2000 and 5–2000 ng/mL for DOC in plasma and urine, respectively, which were performed and measured within one day. Next, the ratios of the DOC peak area to the peak area of the IS were established, and calibration curves, using a linear regression model based on the described parameters as a function of DOC concentration, were calculated. The obtained data, reported in Table 2, confirm that the proposed UA-DLLME-LC–MS/MS method provides linearity in the range of 2.5–2000 ng/mL for plasma and 5–2000 ng/mL for urine samples, with a correlation coefficient > 0.9997. In the case of urine samples, linearity was confirmed in a wider range than that reported in the literature [19].

Additional tests were carried out to determine whether the developed method can be applied to analyze samples that have been diluted to concentrations beyond the upper limit of linearity. For this investigation, samples at a concentration of 3000, 4000, and 5000 ng/mL (each in six repetitions) were prepared and diluted 4-fold in a mixture of ACN:water:FA (8:2:0.1, *v*/*v*/*v*). The results of these tests revealed precision and accuracy in the ranges of 2.91–5.87% and 94.97–106.42%, confirming their compliance with the standards set forth for bioanalytical methods. Therefore, the proposed UA-DLLME-LC–MS/MS method for the quantification of DOC in plasma samples can be applied in the upper confirmed linearity range, which is wider than those reported for previous LC protocols [12,14,15,16,18,23].

#### 3.2.3. Limit of Detection and Limit of Quantification

The method’s limit of detection (LOD) was defined as the concentration of DOC in plasma and urine samples at which the signal to noise level (S/N) was 3:1 (n = 6). In this research, the LODs for the plasma and urine samples were 1 ng/mL and 2.5 ng/mL, respectively (Table 2). The lower limit of quantification (LLOQ) was defined as the lowest concentration of DOC in plasma and urine samples wherein the signal to noise level (S/N) was 10:1, and precision and accuracy were <15% and between 80–120%, respectively. The LLOQs obtained for plasma and urine were 2.5 ng/mL and 5 ng/mL, respectively (Table 3). In both cases, the obtained values were the first points on the calibration curves for DOC determination in plasma and urine samples. The calculated LODs and LLOQs were lower than those previously reported in the literature [12,13,14,15,16,23].

#### 3.2.4. Accuracy and Precision

Accuracy and precision were evaluated by applying the proposed method to analyze plasma and urine samples spiked with DOC at low (LQC: 50 ng/mL for plasma and 250 ng/mL for urine samples), medium (MQC: 750 ng/mL for both matrices), and high (HQC: 1500 ng/mL for both matrices) concentrations, and IS at concentrations of 100 ng/mL (plasma) and 50 ng/mL (urine). To measure intra-day and inter-day precision and accuracy, each sample was prepared in six replicates and analyzed on the same day and between days within 2 months of preparation. Accuracy was defined as the recovery (%) of DOC versus the final concentration of DOC in enriched plasma and urine samples, while precision was defined based on the relative standard deviation (RSD (%)). The data relating to the method’s intra-day and inter-day accuracy and precision are presented in Table 3.

As shown in Table 3, the intra-day and inter-day accuracies for the determination of DOC in plasma and urine samples were in the ranges of 90.87–100.69% and 96.70–105.53%, while the intra-day and inter-day precisions were within 2.28–6.45% and 2.64–5.18% for plasma and 3.95–6.25% and 0.98–3.33% for urine. The obtained data for all QC samples confirm that the proposed method satisfies the generally accepted criteria for bioanalytical method validation with respect to accuracy and precision.

#### 3.2.5. Carry Over and Recovery Results 

Carry over and matrix effects were evaluated by applying the optimized UA-DLLME-LC–MS/MS method to analyze samples containing DOC and PAC at concentrations of 2000 and 100 ng/mL and blank samples without analytes, each in triplicate. The blank samples were analyzed after the samples were enriched with DOC and IS. The obtained data (data not shown) indicate that carry over does not affect the method’s ability to quantify DOC in plasma and urine samples.

The absolute recovery of DOC was measured by applying the proposed method to analyze urine and plasma samples containing the analytes at concentrations of 500 ng/mL and 1000 ng/mL, and IS at concentrations 100 and 50 ng/mL for plasma and urine, respectively. Each of the samples was prepared and analyzed in triplicate. Next, the signals from the samples enriched with DOC and PAC prior to extraction were compared with the signals from the same samples obtained after extraction. The mean efficiencies for DOC and PAC at concentrations of 500 ng/mL in plasma samples were 40.96 ± 2.06% and 41.98 ± 2.11%, respectively, while at a concentration of 1000 ng/mL, the mean efficiencies for DOC and PAC were 40.50 ± 2.05% and 42.30 ± 1.38%, respectively. In the urine samples, the proposed method had mean recoveries of 81.96 ± 3.75% and 80.70 ± 2.59% for DOC and PAC, respectively, when present at concentrations of 500 ng/mL. At a concentration of 1000 ng/mL in urine, the method provided recoveries of 80.84 ± 3.28% for DOC and 79.55 ± 1.02% for PAC.

#### 3.2.6. Stability Study

The stability of DOC in plasma and urine samples was tested at three QC concentration: low, medium, and high. Each QC sample was prepared in triplicate and analyzed after being stored under different conditions; namely, short-term storage (25 °C for 8 h), long-term storage (−80 °C for 2 months), after three freeze/thaw cycles (−80 °C to room temperature), and after post-preparative storage (4 °C for 24 h). The resultant data were compared with the results obtained from the analysis of fresh QC samples. The obtained data confirmed that DOC and the IS remained stable in plasma and urine samples across the different storage conditions (Table S1).

### 3.3. Application to Real Samples

**Figure 2:**
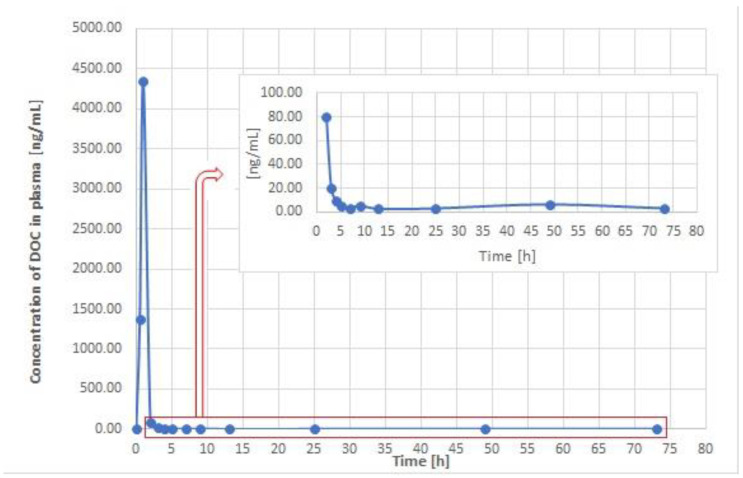
DOC concentration profiles in time intervals for plasma samples from a 12-year-old cardiac AS patient after a 1 h IV infusion of DOC at a dose of 45 mg (30 mg/m^2^).

**Figure 3:**
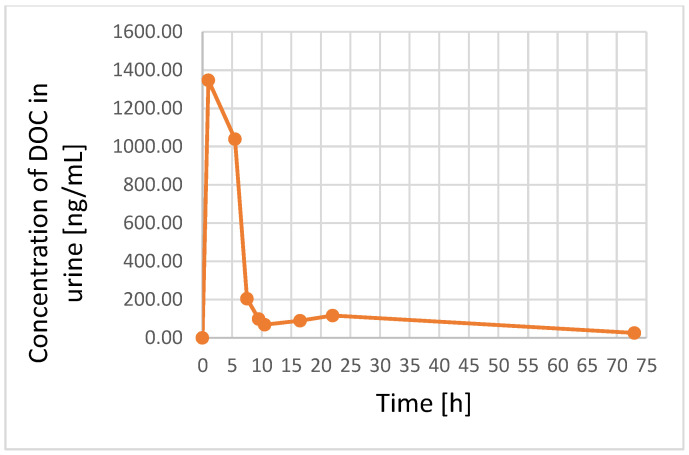
DOC concentration profiles in time intervals for urine samples from a 12-year-old cardiac AS patient after a 1 h IV infusion of DOC at a dose of 45 mg (30 mg/m^2^).

The UA-DLLME-LC–MS/MS method was applied to profile the concentration of DOC in plasma and urine samples obtained from a 12-year-old male cancer patient who was receiving a 45 mg dose of DOC intravenously for 1 h every 3 weeks (30 mg/m^2^) (Section 2.2 and Section 2.7). The chromatograms presented in Figure S4 show the levels of DOC in the plasma and urine samples obtained at the end of the IV administration of DOC, and the signal of IS which was added to the tested samples at the level of of 100 and 50 ng/mL, respectively. The concentrations of DOC in the patient’s plasma and urine samples were calculated using the calibration curves reported in Table 2. The DOC concentration profiles obtained for the plasma and urine samples after IV administration are shown in Figure 2 and Figure 3, respectively.

The obtained profiles for DOC revealed that, for both the plasma and urine samples, the maximum concentration of DOC was reached just after IV administration, and it remained at that level throughout the 1 h treatment. In the case of the plasma samples, C_max_ was 4336.51 ± 315.57 ng/mL at 1 h. The results further showed that DOC levels decreased up to the 8 h mark, with a minimal increase being observed at about 10 h followed by further decline up to 15 h. After that point, the level of DOC slightly increased once again at about 49 h, and then finally decreased to 3.01 ± 0.98 ng/mL at 73 h (Figure 2).

In the urine samples, the maximum concentration of DOC was observed at the end of infusion, with levels measuring 1347.55 ± 130.29 ng/mL. After that time-point, the concentration of DOC decreased to 1039.62 ± 80.56 ng/mL and 205.36 ± 10.97 ng/mL at 4.5 h and 6.5 h post-infusion, respectively. Similar to the profiles obtained for the plasma samples, the level of DOC increased slightly at 22 h before it decreased once again. At 73 h, the concentration of DOC in urine was 26.08 ± 10.33 ng/mL (Figure 3). The literature contains only one study wherein DOC levels were determined in urine samples after oral administration; however, there is an absence of studies detailing the profiling of DOC in urine samples. In the lone study focusing on the determination of DOC in urine, the patient received a 60 mg dose of DOC orally in combination with ritonavir, with findings showing that about 2.5% of the dose remained detectable in the patient’s urine 7–24 h after administration [19]. In our study, C_max_ in urine samples was 1347.55 ± 130.29 ng/mL, which was lower than the DOC levels observed in the plasma samples. Moreover, the determined value in the present study was higher than the per cent of the daily dose detected after oral administration in the previous study [19]. The observed fluctuations in the DOC concentration in plasma samples at later time-points may reflect the three-compartment model of this drug, which is consistent with previous research [8]. Moreover, the obtained DOC levels could be useful in determining subsequent dosage regimens. These data could also be significant in selecting dose regimens for DOC pharmacotherapy.

The above data show that the concentration of DOC in plasma samples was much higher than in urine samples, which confirms previous observations that only about 5% of the dose is eliminated via the kidneys. Nevertheless, despite the small amount of DOC eliminated via urine, the advantage of using this matrix for drug monitoring is that it is non-invasive compared to the use of bile samples.

The samples used in this work were obtained from a pediatric patient diagnosed with cardiac AS, which is a very rare form of children’s cancer. Given the rarity of this cancer, the availability of data relating to pharmacotherapy and dosing regimens is limited, which makes it challenging to develop appropriate treatment schedules. Therefore, researchers have recommended the development of new approaches to treating this disease, such as multimodal therapy [29]. Until now, there have been no studies examining the application of TDM in pediatric cardiac AS patients receiving DOC-based treatment. Previously, standard treatment schedules have mainly been based on the use of anthracycline drugs in monotherapy and polytherapy, such as VAC (vincristine, doxorubicin, cyclophosphamide) or CAD (cyclophosphamide, doxorubicin, dacarbazine) [30]. Furthermore, the application of CEVAIE (carboplatin, epirubicin, vincristine, actinomycin D, ifosfamide, and etoposide) has also been reported [29]. Although polytherapy frequently results in interactions between the constituent drugs, which leads to undesired side-effects and the risk of toxicity, its synergistic pharmacologic action tends to foster a better response to the applied pharmacotherapy [34]. According to the literature, the use of anthracyclines is associated with cardiotoxicity, which is the main adverse effect limiting their application to a maximum cumulative dose of 500 mg [35]. DOC and PAC have also been used in combination with gemcitabine as a second-line drug in the treatment of sarcomas [36]. The DOC dose described in the literature for adult cardiac AS patients is 25 mg/m^2^ weekly [31], which is very close to the dose received by the pediatric patient in this study (45 mg IV for 1 h (30 mg/m^2^)) (Section 2.7). Moreover, based on prior studies focusing on adult patients, the maximum level of DOC in plasma was 1036 ± 228 ng/mL following a dose of 25–35 mg/m^2^ administered over a 30 min period [17]. DOC has also been administered in combination with oxaliplatin and capecitabine in a Phase I clinical trial. On the other hand, 2035 ± 477 ng/mL of DOC was measured after a 75 mg/m^2^ dose was administered over a 2 h period [23]. In another study, the maximal levels of DOC in plasma samples obtained from two patients received this drug at a dose of 100 mg administered over 1 hr were 120.43 µg/L and 176.17 µg/L [18]. The same dosing schedule (i.e., 100 mg IV for 1 h) was applied in another study, with the detectable amounts at the end of treatment equaling 2060 ng/mL [37]. This maximum concentration of DOC was approximately seventeen times higher than the C_max_ obtained in the previous study (120.43 µg/L) [18]. These fluctuations may also be due to variability between patients and/or may be the result of drug interactions, as the patients in each case were receiving polytherapy. However, as mentioned above, there are no data for DOC in plasma and urine samples that can be referenced in comparison to those obtained for the pediatric cardiac AS patient in this study. Thus, in the case of pediatric patients, several other factors, such as age and liver function, should be taken into consideration, as the activity of CYP3A4—and hence liver metabolism—could influence the elimination of DOC from the body [38]. In addition, studies examining the pharmacokinetics of DOC have shown that differences in the composition of plasma proteins, especially acid glycoprotein α1, may impact the level of this drug [8,39,40]. Therefore, to improve the efficiency of treatment, it is crucial to develop a methodology that can be applied in TDM studies to allow the adjustment of the optimal dose of DOC in paediatric oncological patients. As indicated in previous studies, significant differences in the AUC and clearance values were observed among patients receiving DOC [11], which confirms the importance of monitoring DOC levels in body fluids. This research addresses this need by presenting a fast, novel UA-DLLME-LC–MS/MS method that enables the precise quantification of DOC in biological matrices, and that can be applied in pharmacokinetic studies aimed at profiling DOC levels in both plasma and urine samples.

To sum up, the developed DLLME-LC–MS/MS protocol provides a viable alternative to the standard methods currently being used for the TDM of DOC in clinical practice. This is critical, as the ability to precisely monitor DOC levels and differences in the efficiency or toxicity of an anticancer therapy can provide a better understanding of the pharmacokinetics of this drug and could also help to produce more accurate dosing information for use during multimodal therapy.

## 4. Conclusions

This paper documented the development of a novel UA-DLLME-LC–MS/MS protocol for profiling DOC concentrations in plasma and urine samples from a pediatric patient. In addition, this work presented and tested an entirely new sample-preparation approach based on UA-DLLME for efficient isolation of DOC from different biomatrices. The results indicate that the proposed protocol provides fast and simple extraction, and can be coupled with MS-based methods for the fast and sensitive monitoring of DOC in biofluids as a part of TDM during anticancer therapy. Furthermore, the developed UA-DLLME-LC–MS/MS method fulfils all validation requirements and is suitable for the monitoring of DOC in plasma and urine samples obtained from a pediatric patient with cardiac AS. The obtained concentration time profiles of DOC demonstrated the proposed method’s practical applicability. While the results indicated that the concentration of DOC was significantly higher in the plasma samples than in the urine samples, a three-compartment model of DOC pharmacokinetics was observed for both matrices. Moreover, concentrations of DOC were detected in both matrices days after the IV administration of a 35 mg/m^2^ dose. To the best of our knowledge, prior reports have only focused on different strategies for treating this very rare disease in children, with no research investigating the TDM of DOC during its use in pharmacotherapy in pediatric patients. The excellent speed and precision offered by the proposed UA-DLLME-LC–MS/MS protocol may improve the general approach to actual oncological strategies, particularly with respect to TDM among pediatric patients in clinical oncology units.

## Figures and Tables

**Table 1:** Recoveries of DOC and PAC at a concentration of 1000 ng/mL from 1 mL PBS after using different sample preparation (n = 3).

**The Recovery of DOC [%]**												
(*v*/*v*)	DChM:MeOH			DChM:EtOH			Chl:MeOH			Chl:EtOH		
	AVG	SD	RSD [%]	AVG	SD	RSD [%]	AVG	SD	RSD [%]	AVG	SD	RSD [%]
4:6	75.09	6.65	8.86	72.53	4.52	6.23	79.86	5.99	7.50	95.00	4.89	5.15
3:7	78.19	4.88	6.24	71.63	5.99	8.36	88.08	6.13	6.96	93.63	5.21	5.56
2:8	69.67	7.05	10.12	74.67	4.41	5.91	82.42	5.69	6.90	93.69	5.75	6.14
**The Recovery of PAC [%]**												
(*v*/*v*)	DChM:MeOH			DChM:EtOH			Chl:MeOH			Chl:EtOH		
	AVG	SD	RSD [%]	AVG	SD	RSD [%]	AVG	SD	RSD [%]	AVG	SD	RSD [%]
4:6	77.86	7.11	9.13	75.20	6.66	8.86	82.80	6.04	7.29	98.50	4.36	4.43
3:7	81.07	5.06	6.24	74.28	5.87	7.90	91.38	7.06	7.73	97.08	5.77	5.94
2:8	72.24	6.45	8.93	77.43	5.05	6.52	85.46	6.23	7.29	97.14	6.02	6.20

**Table 2:** Validation data for the DLLME-LC–M/MS method in the determination of DOC in plasma and urine samples (n = 6).

Parameters	Plasma Samples	Urine Samples
Linearity (ng/mL)	2.5–2000	5–2000
Equation parameter		
Slope	0.0013 ± 0.0000067	0.0025 ± 0.000013
Intercept	−0.0007 ± 0.006	−0.0063 ± 0.012
Correlation coefficient (R^2^)	0.9998	0.9997
LOD (ng/mL)	1	2.5

**Table 3:** Accuracy and precision for the determination of DOC in plasma and urine samples (n = 6).

	Plasma Samples				Urine Samples			
	Concentration (ng/mL)				Concentration (ng/mL)			
	Spiked	Found (Mean ± SD)	Precision (RSD %)	Accuracy (%)	Spiked (ng/mL)	Found (Mean ± SD)	Precision (RSD %)	Accuracy (%)
Intra-day (n = 6)								
LLOQ	2.5	2.71 ± 0.25	9.42	108.21	5	4.92 ± 0.42	8.60	98.40
LQC	50	45.44 ± 2.93	6.45	90.87	250	263.83 ± 16.49	6.25	105.53
MQC	750	745.37 ± 21.85	2.93	99.38	750	731.40 ± 33.47	4.58	97.52
HQC	1500	1460.54 ± 33.32	2.28	97.37	1500	1495.80 ± 59.11	3.95	99.72
Inter-day (n = 6)								
LLOQ	2.5	2.36 ± 0.26	11.09	94.27	5	4.84 ± 0.55	11.45	96.70
LQC	50	46.78 ± 2.42	5.18	93.56	250	245.54 ± 8.18	3.33	98.21
MQC	750	755.17 ± 25.03	3.31	100.69	750	738.34 ± 16.70	2.26	98.44
HQC	1500	1488.25 ± 39.26	2.64	99.22	1500	1495.47 ± 14.65	0.98	99.69

## Data Availability

Not applicable.

## Supplementary Materials

The following supporting information can be downloaded at: https://www.mdpi.com/article/10.3390/pharmaceutics15041255/s1, Figure S1: Structure of docetaxel (DOC) (A) and paclitaxel (PAC) (B). DOC: Formula: C_43_H_53_NO_14_, Molar mass: 807.879 g/mol; PAC: Formula: C_47_H_51_NO_14,_ Molar mass: 853.918 g/mol; Figure S2: The peak area of DOC (A) and PAC (B) calculated for plasma samples containing DOC and PAC (IS) at the concentration of 1 µg/mL and 100 ng/mL, respectively, tested at different times of sonication (without sonication, 0.5 min, 1, 2, and 5 min); Figure S3: Chromatograms of blank human plasma (1A, 1B) and urine (2A, 2B) samples without DOC and PAC addition, respectively. Chromatograms of plasma samples after DLLME-LC–MS/MS analysis of DOC (250 ng/mL) (1C) and PAC (IS) (100 ng/mL) (1D). Chromatograms of urine samples after DLLME-LC–MS/MS analysis of DOC (250 ng/mL) (2C) and PAC (IS) (50 ng/mL) (2D). MRM transitions for DOC: 808.2 *m*/*z* -> 181.9 *m*/*z*, and for PAC: 854.2 *m*/*z* -> 285.7 *m*/*z*; Figure S4: Chromatograms of DOC in plasma (1A) and urine (2A) samples after the administration of a 35 mg/m^2^ dose of this drug administered to pediatric cancer patient spiked with IS (100 ng/mL) (1B) and (50 ng/mL) (2B), respectively. MRM transitions for DOC: 808.2 *m*/*z* -> 181.9 *m*/*z*, and for PAC: 854.2 *m*/*z* -> 285.7 *m*/*z*; Table S1: Stability of DOC in plasma and urine samples under various conditions (mean ± SD, n = 3).
