# Supplementary material for: Profiling Docetaxel in Plasma and Urine Samples from a Pediatric Cancer Patient Using Ultrasound-Assisted Dispersive Liquid–Liquid Microextraction Combined with LC–MS/MS
Source: Pharmaceutics. 2023 Apr 17;15(4):1255. doi: 10.3390/pharmaceutics15041255 (PMC10143245; doi:10.3390/pharmaceutics15041255)
Supplement: Supplementary file 1 [file pharmaceutics-15-01255-s001.zip › pharmaceutics-2326960-supplementary.pdf]

Supplementary materials

Profiling Docetaxel in Plasma and Urine Samples from a Pediatric Cancer Patient Using Ultrasound-Assisted Dispersive Liquid–Liquid Microextraction Combined with LC–MS/MS

Olga Maliszewska [1,2], Anna Roszkowska [2], Marcin Lipiński [3], Natalia Treder [1,2], Ilona Olędzka [2], Piotr Kowalski [2], Tomasz Bączek [2], Ewa Bień [4], Małgorzata Anna Krawczyk [4] and Alina Plenis [1,*]

[1] Department of Analytical Chemistry, Medical University of Gdansk, 80-416 Gdańsk, Poland
[2] Department of Pharmaceutical Chemistry, Medical University of Gdansk, 80-416 Gdańsk, Poland
[3] Department of Pharmaceutical Biochemistry, Medical University of Gdansk, 80-211 Gdańsk, Poland
[4] Department of Pediatrics, Hematology and Oncology, Medical University of Gdansk, 80-211 Gdańsk, Poland
* Correspondence: aplenis@gumed.edu.pl; Tel.: +48-58-349-10-96; Fax: +48-58-349-15-24

Contents:

A
B

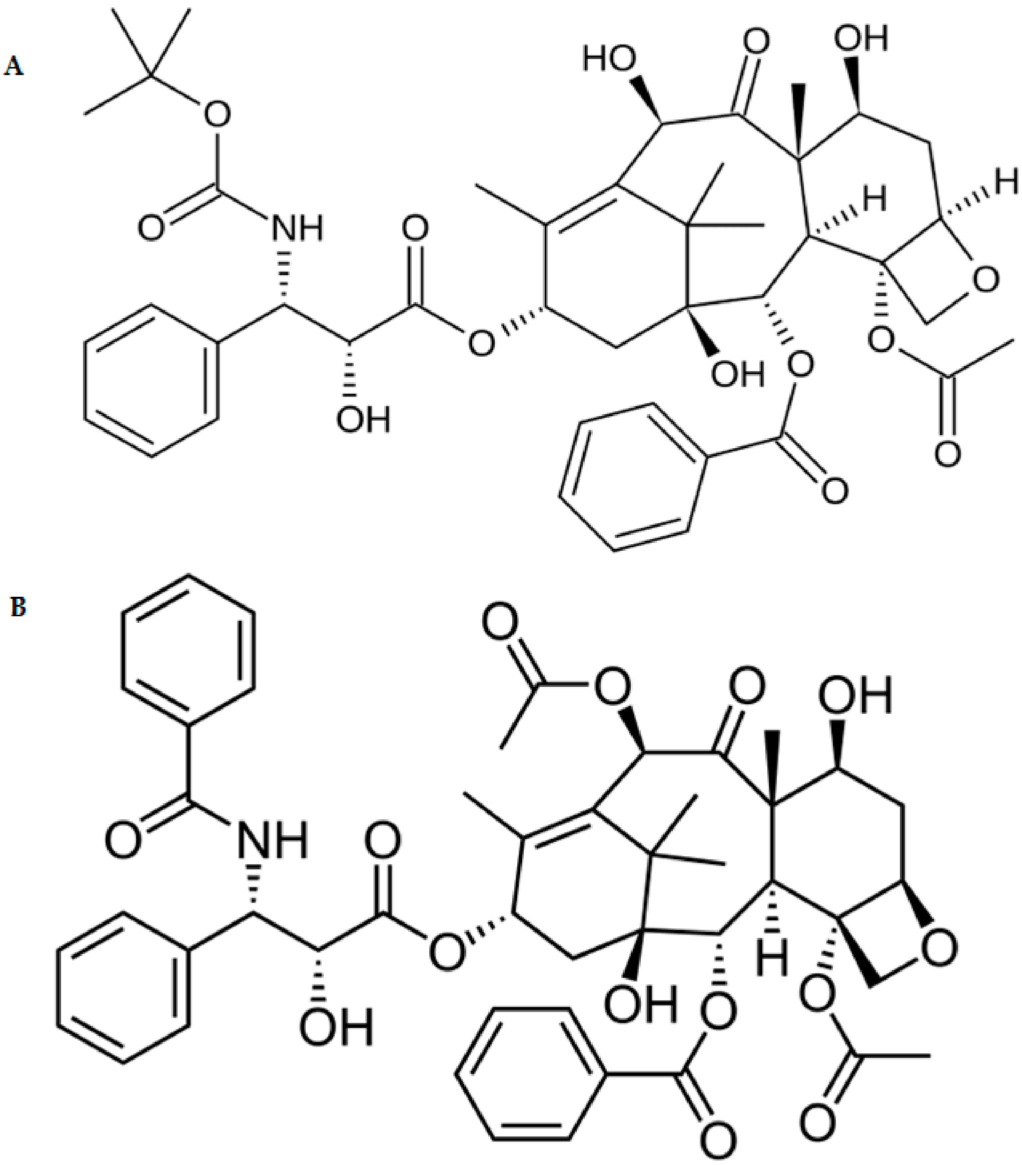

Figure S1. Structure of docetaxel (DOC) (**A**) and paclitaxel (PAC) (**B**). DOC: Formula: $C_{43}H_{53}NO_{14}$, Molar mass: 807.879 g/mol; PAC: Formula: $C_{47}H_{51}NO_{14}$, Molar mass: 853.918 g/mol.

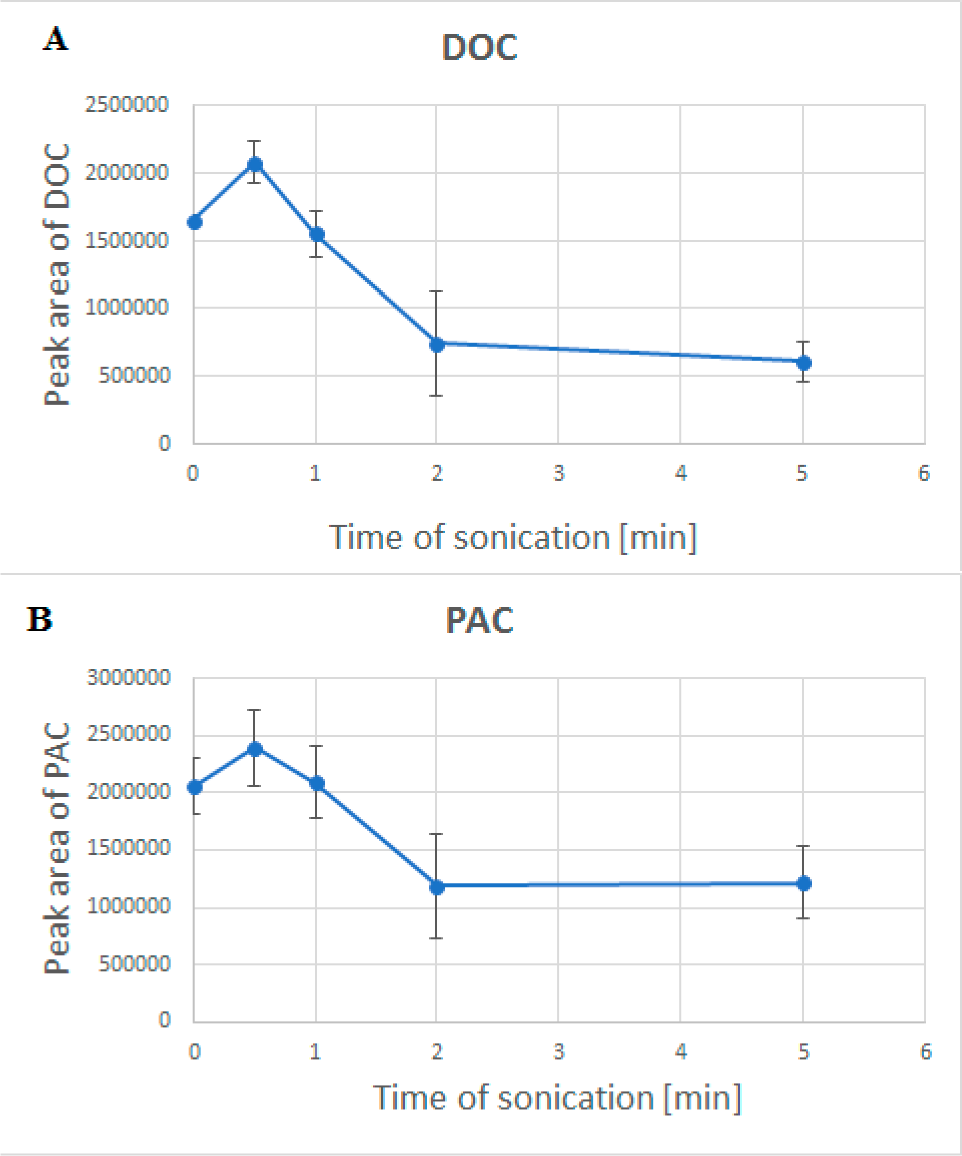

Figure S2. The peak area of DOC (A) and PAC (B) calculated for plasma samples containing DOC and PAC (IS) at the concentration of 1 μg/mL and 100 ng/mL, respectively, tested at different times of sonication (without sonication, 0.5 min, 1, 2 and 5 min).

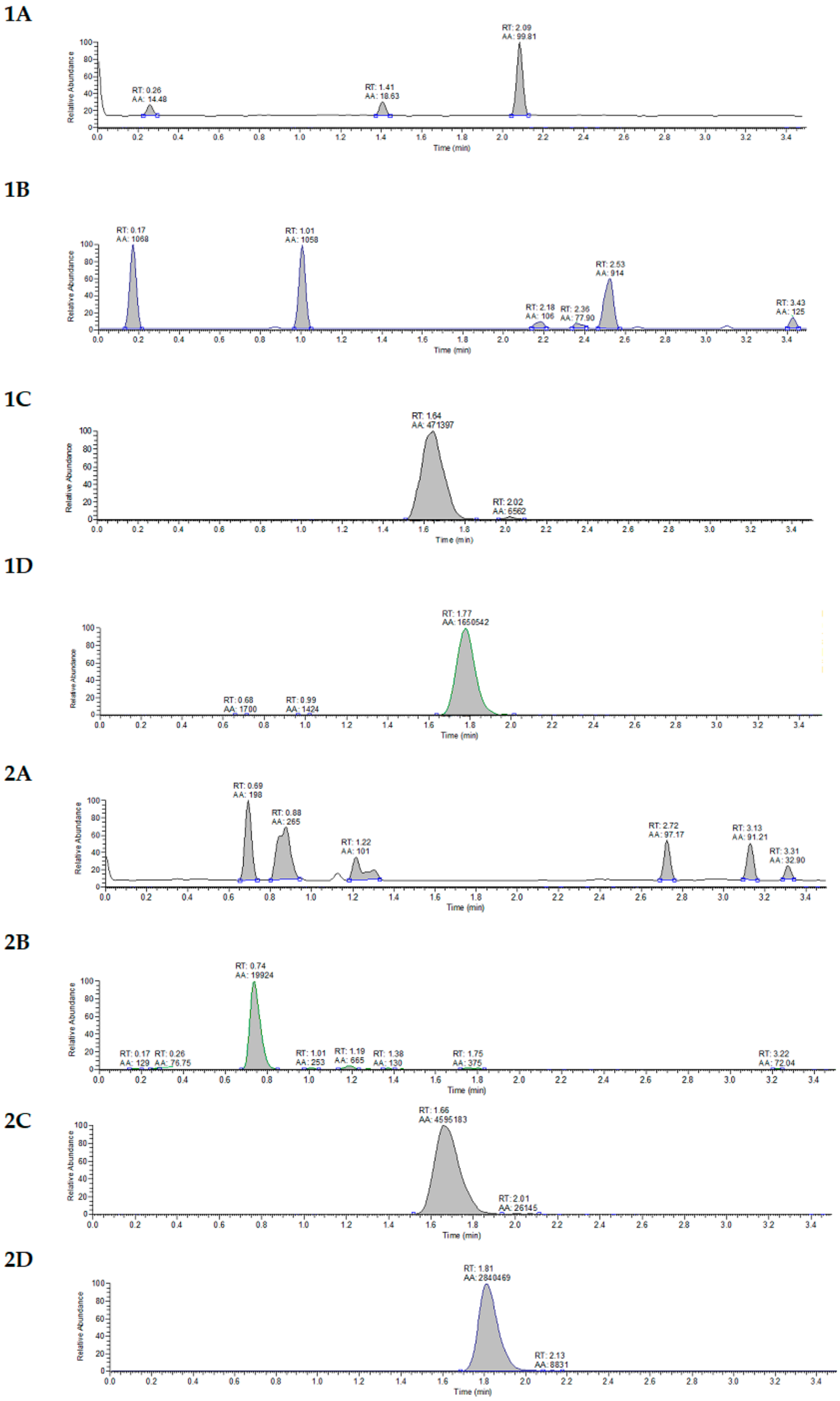

Figure S3. Chromatograms of blank human plasma (**1A, 1B**) and urine (**2A, 2B**) samples without DOC and PAC addition, respectively. Chromatograms of plasma samples after DLLME-LC-MS/MS analysis of DOC (250 ng/mL) (**1C**) and PAC (IS) (100 ng/mL) (**1D**). Chromatograms of urine samples after DLLME-LC-MS/MS analysis of DOC (250 ng/mL) (**2C**) and PAC (IS) (50 ng/mL) (**2D**). MRM transitions for DOC: 808.2 m/z -> 181.9 m/z, and for PAC: 854.2 m/z -> 285.7 m/z.

1A

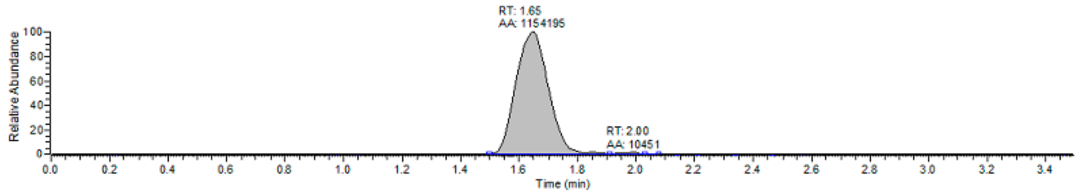

1B

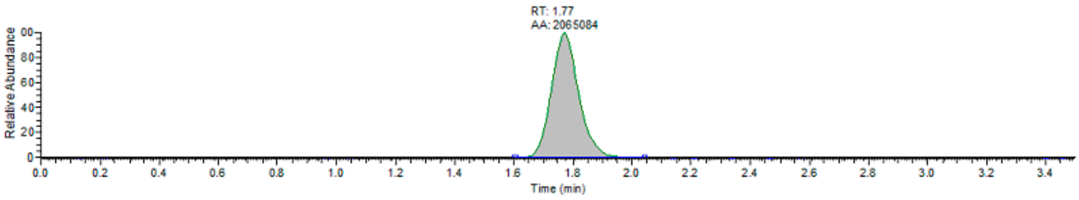

2 A

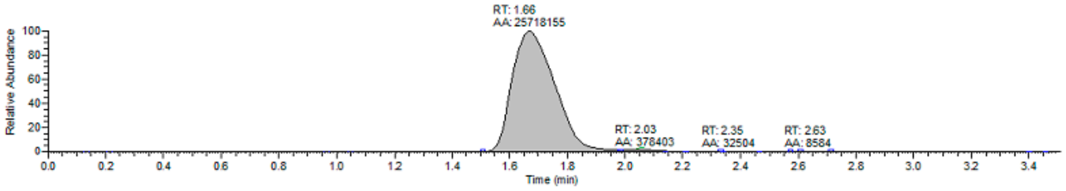

2 B

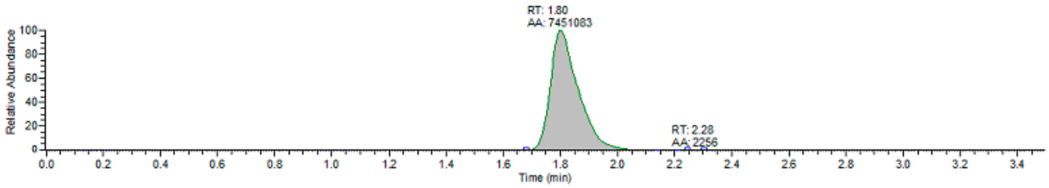

Figure S4. Chromatograms of DOC in plasma (**1A**) and urine (**2A**) samples after the administration of a 35 mg/m^2 dose of this drug administered to pediatric cancer patient spiked with IS (100 ng/mL) (**1B**) and (50 ng/mL) (**2B**), respectively. MRM transitions for DOC: 808.2 m/z -> 181.9 m/z, and for PAC: 854.2 m/z -> 285.7 m/z.

Table S1. Stability of DOC in plasma and urine samples under various conditions (mean ± SD, n = 3).

**Storage Conditions**	**QC**	**Conc. added (ng/mL)**	**Found* (ng/mL)**	**Precision RSD (%)**	**Accuracy (%)**
		Plasma			
Short-term stability (25°C, 8 h)	LQC	50	52.57 ± 2.92	5.55	105.14
	MQC	750	741.64 ± 24.49	3.30	98.88
	HQC	1500	1484.26 ± 47.55	3.20	98.95
Long-term stability (-80°C, 2 months) stability	LQC	50	45.6 ± 4.12	9.03	91.12
	MQC	750	759.92 ± 36.89	5.24	101.32
	HQC	1500	1467.57 ± 71.09	4.84	96.7
Three freeze-thaw cycles stability	LQC	50	47.83 ± 4.21	8.80	95.66
	MQC	750	769.67 ± 44.66	5.80	102.62
	HQC	1500	1524.26 ± 86.28	5.66	101.62
Post-preparative storage (4°C, 24 h)	LQC	50	47.63 ± 2.32	4.87	95.26
	MQC	750	749.22 ± 21.58	2.88	99.89
	HQC	1500	1507.40 ± 38.42	2.54	100.49
			Urine		
Short-term stability (25°C, 8 h)	LQC	250	245.51 ± 12.02	4.89	91.02
	MQC	750	716.83 ± 44.28	6.17	95.57
	HQC	1500	1514.05 ± 62.94	4.16	100.94
Long-term stability (-80°C, 2 months) stability	LQC	250	257.22 ± 15.37	5.97	102.88
	MQC	750	771.66 ± 57.99	7.51	102.89
	HQC	1500	1461.33 ± 88.04	6.02	97.42
Three freeze-thaw cycles stability	LQC	250	227.55 ± 19.37	8.51	91.02
	MQC	750	769.06 ± 57.99	7.54	102.54
	HQC	1500	1485.25 ± 71.18	4.79	99.02
Post-preparative storage (4°C, 24 h)	LQC	250	244.52 ± 9.02	3.69	97.81
	MQC	750	742.38 ± 32.46	4.37	98.98
	HQC	1500	1474.52 ± 38.09	2.58	96.7

QC - quality control; LQC – low quality control; MQC – middle quality control; HQC - high low quality control
